# Endoscopic repair of duodenal perforations, a scoping review

**DOI:** 10.1007/s00464-024-11133-x

**Published:** 2024-08-14

**Authors:** Jennifer Williams, Hansa Joshi, Michael Schwartz, Ami Kalola, Alvin Mercado, Benjamin Saracco, Amanda Adams, Adib Chaaya, Daniel Baik, Adam Elfant, Young Ki Hong

**Affiliations:** 1 Department of Surgery, Cooper University Hospital, 3 Cooper Plaza, Suite 411, Camden, NJ 08103 USA; 2 Department of Gastroenterology, Cooper University Hospital, Camden, NJ USA; 3 Cooper Medical School of Rowan University, Camden, NJ USA

**Keywords:** Duodenum, Perforation, Endoscopy, Repair, Surgery, Review

## Abstract

**Background:**

There is a discrepancy in the surgical and endoscopic literature for managing duodenal perforations. Although often managed conservatively, surgical repair is the standard treatment for duodenal perforations. This contrasts with the gastroenterology literature, which now recommends endoscopic repair of duodenal perforations, which are more frequently iatrogenic from the growing field of advanced endoscopic procedures. This study aims to provide a scoping review to summarize the current literature content and quality on endoscopic repair of duodenal perforations.

**Methods:**

The protocol for performing this scoping review was outlined by the Joanna Briggs Institute. All studies that reported primary outcomes of patients who had undergone endoscopic repair of duodenal perforations before February 2022, regardless of perforation etiology or repair type were reviewed, with studies after 1999 meeting inclusion criteria. The study excluded articles that did not report clinical outcomes of endoscopic repair, articles that did not describe where in the gastrointestinal tract the endoscopic repair occurred, pediatric patients, and animal studies.

**Results:**

7606 abstracts were screened, with 474 full articles reviewed and 152 studies met inclusion criteria. 560 patients had duodenal perforations repaired endoscopically, with a technical success rate of 90.4% and a survival rate of 86.7%. Most of these perforations (74.5%) were iatrogenic from endoscopic procedures or surgery. Only one randomized control trial (RCT) was found, and 53% of studies were case reports.

**Conclusion:**

These results suggest that endoscopic repair could emerge as a viable first-line treatment for duodenal perforation and highlight the need for more high-quality research in this topic.

**Supplementary Information:**

The online version contains supplementary material available at 10.1007/s00464-024-11133-x.

Duodenal perforations are rare but devastating. There are many causes of duodenal perforations, including peptic ulcer disease, iatrogenic injury, and trauma [1]. Iatrogenic injury is often associated with endoscopic retrograde cholangiopancreatography (ERCP). The Stapfer classification is used to divide perforations into Type I, lateral or medial wall duodenal perforation; type II, perivaterian injuries; type III, distal bile duct injuries related to guidewire-basket instrumentation; and type IV, retroperitoneal air alone [2]. Perforation can be detected during endoscopic procedures with direct visualization or post-procedure with imaging.

A prior literature review from 2000 to 2014 found that during ERCP, endoscopic sphincterotomy was found to be responsible for 41% of perforations, insertion and manipulations of the endoscope for 26%, guidewire for 15%, dilation of strictures for 3%, other instruments for 4%, stent insertion and migration for 2%, and in 7% of cases the etiology was unknown [3].

Surgical management of perforations includes performing an omental patch or reconstructive surgery, including a duodenoduodenostomy, Roux-en-Y duodenojejunostomy, or Billroth II operation [4]. The mortality rate from a duodenal perforation can range from 8 to 25% [5].

Over the past few years, development of novel devices has allowed some of these perforations to be repaired endoscopically. Repairs have been successful using endoclips, larger over-the-scope clips (OTSC), and endoscopic suturing devices [6]. In the case of Type 1 perforations, placement of OTSC at the time of procedure led to good outcomes, shorter hospital stays, and prevented the need for subsequent surgery, and is generally used in perforations up to 2 cm in size [7]. Endoscopic repair of perforations is a strongly emerging method, however gaining perspective on outcomes has been difficult as there are mostly case reports in the literature. There are no consensus guidelines on which technique is preferred or the size of the defect appropriate to repair endoscopically, however the gastroenterology literature recommends repairing iatrogenic perforations endoscopically [8]. Despite this, there has been no consensus in the surgical literature for endoscopic management, as the standard treatments are usually conservative, utilizing diet restriction and antibiotics, or surgical repair. Our study sought to address utility of endoscopic repair in treating duodenal perforations, by reviewing the present literature.

## Methods

### Review type

A scoping review was performed to gain a broad view of the current literature content and quality on endoscopic repair of duodenal perforations. The Joanna Briggs Institute outlined the protocol for achieving this scoping review. A systematic review was considered; however, we aimed to gather information and evaluate the gaps in the literature rather than compare groups, making a scoping review more appropriate.

### Inclusion criteria

All studies that reported primary outcomes of patients who had undergone endoscopic repair of duodenal perforations were included. Case reports and case series were included in order to do a comprehensive review of the literature for this scoping study. No language, country of origin, or publication type restrictions were used. Adult patients who underwent endoscopic repair of duodenal perforations were included regardless of the cause of perforation or type of endoscopic repair technique.

### Exclusion criteria

The study excluded articles that did not report clinical outcomes of endoscopic repair, studies that did not describe where in the gastrointestinal (GI) tract the endoscopic repair occurred, pediatric patients, animal studies, pure endoscopic evaluation, or removal of foreign bodies without repair. It also excluded studies in which primary data was unavailable or concurrent surgical and endoscopic procedures were used for repairing duodenal perforations. Abstracts or posters without full articles were also excluded.

### Primary outcomes

The primary outcomes evaluated were the technical success rate of endoscopic repair, major complications, and survival.

### Secondary outcomes

The secondary outcomes were minor complications, such as stent migration, post-procedure abdominal pain or localized peritonitis, delayed healing, bleeding without hemodynamic changes, or stricture, re-intervention, whether endoscopically or surgically, and length of stay.

### Information sources

To identify potentially relevant documents, the following bibliographic databases were searched for all dates before February 2022: Medline using the Ovid interface, Embase, Google Scholar, and Cochrane Central Register of Controlled Trials. The Web of Science Conference Proceedings Citation Index was searched to identify potential gray literature.

### Search strategy

The search strategies were created and drafted in consultation with experienced medical librarians, and further refined through team discussion. Search strategies were peer-reviewed by a second medical librarian before being finalized. Gray literature was identified by searching for conference proceedings using the Web of Science interface and an advanced Google Scholar search. All the search strategies used in this study are available in the protocol (Appendix 1). The final search results were exported into Rayyan QCRI review blinded screening software, and duplicates were removed using EndNote citation management software, followed by a manual review by the librarian research team members. Full-text articles were uploaded into Rayyan after the initial title/abstract screen for all remaining articles.

Two reviewers screened results in Rayyan by title and abstract. After the blinded screening of the titles and abstracts of the articles, disagreements were tie-broken by the study’s principal investigator. Reviewers then sequentially evaluated the remaining titles, abstracts, and full text of all publications identified by our searches for potentially relevant publications. As a team, we resolved any additional disagreements on study selection and data extraction, if needed.

### Study selection

Deduplication software (DeDupe-UI) removed duplicates from the search results. Study personnel then manually reviewed articles flagged as duplicates by Rayyan. At least two team members, comprised of an attending surgeon, surgery residents, gastroenterology fellow, and medical students, did a blinded screening of each abstract, and when there was a conflict, an additional team member served as a tiebreaker for disagreements. All abstracts for consideration had the full-text articles reviewed for inclusion.

### Data collection process

A spreadsheet was used to collect the data from all included articles. The following data was collected: publication type, country, language, start and end dates of data collection, number of patients with duodenal perforations repaired endoscopically, technique for repair, indication for endoscopic repair/ method of perforation, method of diagnosis, success rate, size of perforation, minor complications, major complications, survival, re-intervention, length of stay, academic vs community hospital, and article type. The risk of bias was assessed using the Joanna Briggs Institute (JBI) 2017 Critical Appraisal Checklist.

### Case reports

The review included 8 points from the JBI case report checklist (Appendix 2) [9]. The authors used previous standards for good (80–100% of available points), fair (50–79% of available points), and poor quality (0–49% of available points) [10].

### Case series

The review included 10 points from the JBI case series checklist (Appendix 3) [9]. The authors used previous standards for good (80–100% of available points), fair (50–79% of available points), and poor quality (0–49% of available points) [10].

### Clinical trials

Quality was evaluated with the 11-point Jadad scale (Appendix 4) [11]. The quality of the study was graded as good quality (10–11 points), fair quality (8–9 points), and poor quality (7 or fewer points).

### Statistical analysis

Primary and secondary outcomes were reported as percentages of the patients or studies that reported the results. Comparative statistics were not used as we were not comparing interventions.

## Results

### Screening results

**Fig. 1:**
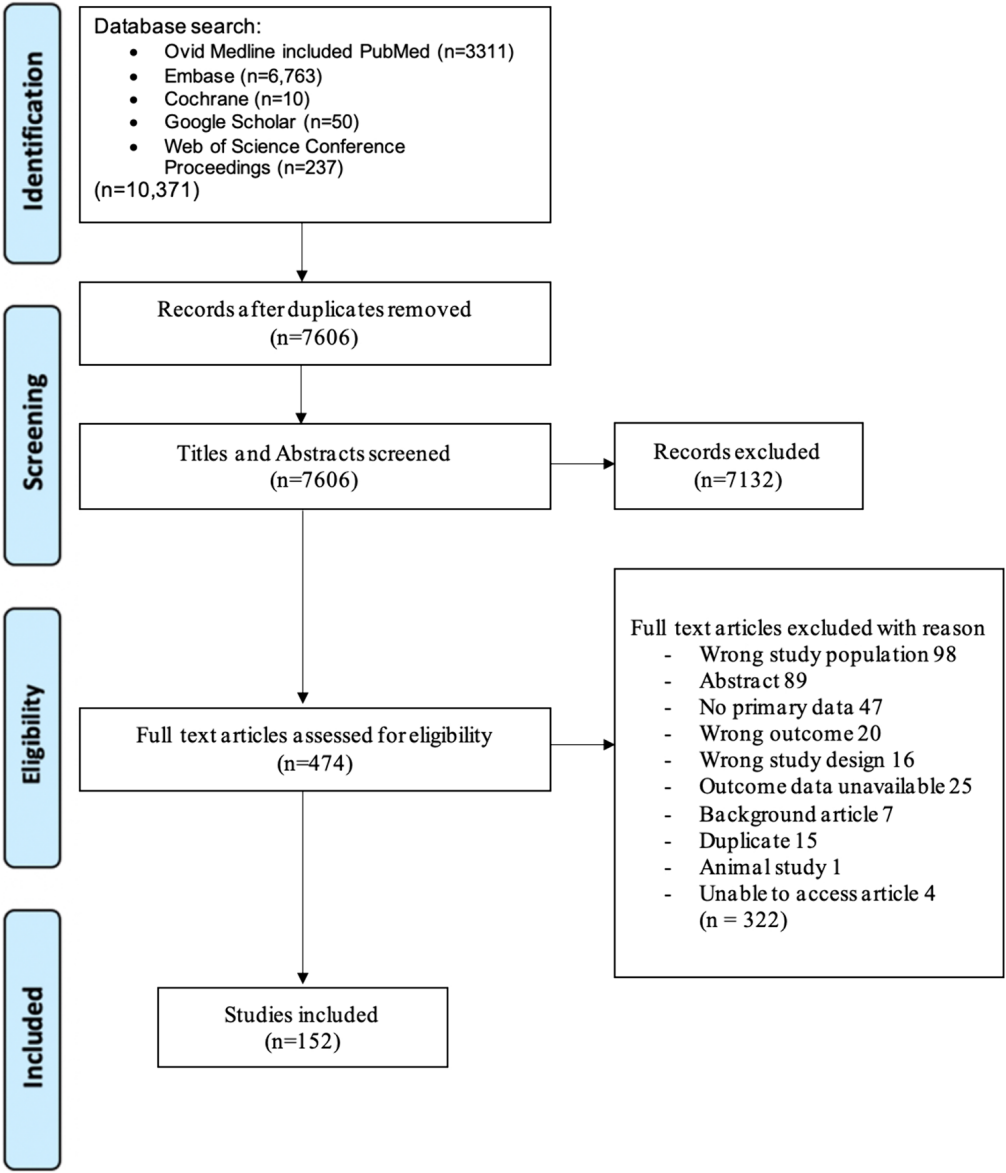
Preferred Reporting Items for Systematic Reviews and Meta-Analysis (PRISMA) flow diagram of study selection

The database search initially resulted in 10,371 studies, which were run through a deduplication software, and 7606 studies remained for abstract and title screening. The studies that did not meet inclusion criteria were excluded (*n* = 7132), leaving 474 articles for full-text review for inclusion. We performed a full-text review of the 474 articles, of which 152 met the inclusion criteria. The articles that were excluded included 98 wrong study population, 89 abstracts or posters without full articles, 47 without primary data, 20 with the wrong outcome, 16 with the incorrect study design, 25 in which the outcome data were unavailable, 7 background articles, 15 duplicates, 1 animal study, and 4 articles unable to be accessed (Fig. 1).

### Study characteristics

Of the 152 studies included, 80 were case reports, 71 were case series, and 1 was a randomized controlled trial. The studies were published from 1999 to 2022. The countries the articles were published from included 1 Argentina, 1 Australia, 1 Belgium, 2 Brazil, 15 China, 1 Columbia, 1 Czech Republic, 7 France, 7 Germany, 3 Greece, 6 India, 1 Ireland, 10 Italy, 23 Japan, 10 Korea, 1 Morocco, 1 Multinational, 1 Netherlands, 1 Norway, 1 Poland, 4 Portugal, 4 Russia, 6 South Korea, 2 Spain, 1 Sweden, 2 Switzerland, 2 Taiwan, 3 Thailand, 7 Turkey, and 27 United States. The languages the studies were published in were: 1 Czech, 143 English, 1 German, 2 Japanese, 3 Korean, and 2 Russian.

### Indications and complications

The indications for endoscopic repair were primarily iatrogenic, which comprised 74.5% of the perforations. These included 1 esophagogastroduodenoscopy, 201 ERCPs, 102 endoscopic mucosal resections (EMR), 16 endoscopic submucosal dissections (ESD), 42 endoscopic ultrasounds (EUS), 33 stent migrations, 12 post-surgical, 1 nasogastric tube, 1 needle-knife dilation of choledochoduodenostomy, 4 duodenoscopies, and 4 listed as iatrogenic perforations from endoscopic procedures. There were 2 perforations due to duodenal diverticulum, 2 from drain migrations, 23 from duodenal ulcers, 4 from fistulas, 3 from foreign bodies, and 2 from trauma. An additional 107 perforations were unspecified in which there were multiple indications in their cohort or they did not fit into one of the categories listed above. 63 studies reported the size of perforations, and sizes ranged from 2 to 48 mm.

### Repair technique

The repair techniques primarily used endoclips or over-the-scope clips (OTSC), which comprised 321 patients; combination methods with endoclips, endoloops, fibrin glue, and/or sutures comprised 96 patients. Stents were used in 90 patients, suture repairs in 21 patients, vacuum therapies in 17 patients, band ligation in 2 patients, fibrin glue in 2 patients, endoloops in 1 patient, helical tacks in 1 patient, and unspecified repair in 9 patients in which 7 comprised of stent or clips without stating which patients had which procedure, and 2 were listed as endoscopic closure but without a specific method.

### Efficacy

The technical success rate of closing perforations was 90.4% (506/560). The index hospital survival rate was 86.7% (477/550), one study did not report survival. Major complications varied; however, they were only described in 25/152 articles. These included sepsis, myocardial infarction, leaks, abscesses, pulmonary embolism, surgery, pancreatitis, and death. Minor complications were described in 18/152 studies. These included abdominal pain, fevers, peritonitis, clip failures, and stent migrations. 42 studies included length of stay, which ranged from 3 to 35 days. 27 studies commented on re-intervention. The most common re-interventions were surgeries, percutaneous drain placements, and repeat endoscopies for dislodged clips or malpositioned stents.

The one randomized controlled study had 12 patients with endoscopic repair of duodenal perforations from ERCPs. The sizes ranged from 5 mm to > 20 mm, and the repairs were done using stents and clips. The immediate technical success rate was 100%, with a survival rate of 91.6% and one death from sepsis. There was 1 patient with a major complication: an abscess requiring drain placement. The average length of stay was 4 days [12].

### Assessment of study validity

Of the case reports, only 28 of 80 (35%) were good quality. Of the rest of the case reports, 39 (49%) were fair quality, and 13 (16%) were poor quality (Appendix 5). Of the case series, only 38 of 71 (54%) were good quality. Of the rest of the case series, 28 (39%) were fair quality and 5 (7%) were poor quality (Appendix 6). One study was a randomized controlled trial that scored 82% (9/11) and was found to be of fair quality (Table 1).

**Table 1:** Study quality of randomized control trials

	Artifon et al. (2015) [12]
Randomized	Yes
Double-blind	No
Description of withdrawals and drop outs	No
Objectives defined	Yes
Outcome measures defined	Yes
Inclusion and exclusion criteria	Yes
Sample size justified	Yes
Intervention description	Yes
Control group	Yes
Method to assess adverse effects described	Yes
Statistical methods described	Yes
Total score	82% (9/11)
Study quality	Fair

## Discussion

We performed a scoping review of all cases of endoscopic repairs of duodenal perforations. Due to the thin wall of the duodenum and difficult location with proximity to the pancreas, liver, and biliary tree, duodenal perforations can be challenging to manage. Most patients with small perforations and no signs of sepsis are managed conservatively with antibiotics, diet restriction, and sometimes percutaneous drainage of abscesses. Any perforation with signs of sepsis have been traditionally managed with surgery. The surgical team has been the primary managing team for these due to the potential for early sepsis.

As advanced endoscopy is growing as a field, there are significantly more iatrogenic perforations due to the increased use of endoscopic resections and treatments for prior surgical conditions such as duodenal lesions or cholangitis, which is now managed primarily endoscopically. The technological advancements allowing for these procedures also have advanced methods for the repair of mucosal and full-thickness defects. For ERCP, the rate of general adverse events is 5–10%, with perforations in 0.3–3.5% of cases [6, 13].

We found 76.4% of perforations repaired endoscopically in the literature were iatrogenic from endoscopic procedures. The remainder of the patients were from duodenal ulcers, diverticulum, fistulas, foreign bodies, or trauma. There was a high technical success rate with endoscopic repair of 90.4% and a mortality rate of 12.4%. One of the common complications, besides sepsis and death, was surgery, which otherwise would be the first-line treatment, followed by percutaneous drain placement for abscesses.

One randomized study compared endoscopic and surgical repair of ERCP-related duodenal perforations. With a study population of only 23, statistical analysis of adverse outcomes was difficult, but endoscopic repair was technically successful for intraperitoneal and retroperitoneal perforations. One patient died of sepsis in the endoscopic group, with no deaths in the surgery group, but the length of stay and cost of the endoscopic group were significantly lower [12].

Although there are no consensus guidelines on when an ERCP-related perforation, let alone any duodenal perforation, can be repaired endoscopically, recent advancements in the field have allowed some Stapfer I and II perforations to be fixed at the time of endoscopy. Recent gastroenterology best practice recommendations now advise that all iatrogenic perforations undergo attempted endoscopic repair with admission and surgical consultation, regardless of the technical success of the endoscopic repair [14]. These determinations need to be made as a collaborative effort with the advanced endoscopists and surgical teams, as considerations such as clinical status, size of perforation, and surgical candidacy need to be evaluated. This collaborative approach requires that patients have presented to centers with advanced endoscopy capabilities and is easiest if they present to the same center that their original procedure was done, as this can prevent fracturing of care.

There are several limitations to our study. The first of which is the nature of a review study, in that although we attempted to use the most effective search strategy, it is possible we missed articles that would have been included otherwise, and similarly, with articles that may have been excluded in the screening process by human error. However, this was attempted to be corrected with two authors screening each abstract for inclusion. The most significant limitation we found is the publication bias of the results. Especially with a large percentage of our included articles being case reports and case series, there is no way to know the actual success rate, as there may be many instances in which endoscopic repair had been attempted but failed and never published. This exemplifies the need for more, higher quality studies on this topic and the need to provide more granular data on size and method of perforation that may be successfully repaired endoscopically.

Endoscopic repair of duodenal perforation may be an option for many perforations if advanced endoscopists are available. This is particularly true if the patient is not an operative candidate, either due to significant comorbidities or a hostile abdomen. This has the potential to redefine the current paradigm in management of duodenal perforations with consultation to advanced endoscopy and surgery being simultaneous, leading to a collaborative approach toward effective and safe repair strategies.

## Conclusion

Endoscopic repair of duodenal perforations is a viable option for first-line treatment. More high-quality studies evaluating endoscopic repair with emphasis on the size and method of repair are needed to provide more granular data and reduce publication bias from case reports and case series as seen in this study. However, the results suggest the surgical dogma should be re-evaluated to involve advanced endoscopists in the comprehensive management of duodenal perforations, particularly in instances where surgical intervention is not a feasible option for the patient.

### Supplementary Information

Below is the link to the electronic supplementary material.

Supplementary file1 (DOCX 18 KB)

Supplementary file2 (DOCX 43 KB)

Supplementary file3 (DOCX 46 KB)
