# Supplementary material for: Endoscopic repair of duodenal perforations, a scoping review
Source: Surg Endosc. 2024 Aug 14;38(9):4839–45. doi: 10.1007/s00464-024-11133-x (PMC11362252; doi:10.1007/s00464-024-11133-x)
Supplement: Supplementary file 1 — Supplementary file1 (DOCX 18 KB) [file 464_2024_11133_MOESM1_ESM.docx]

**Appendix 1:** Search Strategies

Ovid Medline

1. ((gastr* adj5 endo*) or ("esophagogastroduodenoscopies" or "oesophagogastroduodenoscopies" or "oesophagogastroduodenoscopy" or "esophagogastroduodenoscopy" or "endoscopic retrograde cholangiopancreatography" or duoden* or intestin* or "gastro-duodenal" or "stomach ulcer" or "peptic ulcer" or "gastric ulcer")).mp. or (Duodenum/ or Endoscopy, Digestive System/ or Intestinal Perforation/)
2. (perforat* or injur*).mp.
3. 1 AND 2
4. ((duoden* or stomach or gastr* or intestin* or peptic) adj10 (perforat* or injur*)).mp.
5. 3 OR 4
6. (endo* or surg* adj10 (repair* or clos* or manage* or treat*)).mp.
7. (Endoscopy/ or Endoscopes/) and (repair* or clos* or manage* or treat*).ti,ab,kf,kw.
8. 6 OR 7
9. 5 AND 8
10. (perforat* or injur*).ti,ab.
11. 7 AND 8
12. (Animals/ or models, animal/) not Humans/
13. 9 NOT 10

Embase

1. ((duoden* OR stomach OR gastr* OR intestin* OR peptic) NEAR/10 (perforat* OR injur*)) AND endo*:ab,ti,kw AND (repair*:ab,ti,kw OR clos*:ab,ti,kw OR manage*:ab,ti,kw OR treat*:ab,ti,kw)
2. perforat*:ti,ab OR injur*:ti,ab
3. 1 AND 2
4. 3 NOT ([animals]/lim NOT [humans]/lim)

**Appendix 2**: JBI Checklist for Quality of Case Reports

1. Were patient’s demographic characteristics clearly described?
2. Was the patient’s history clearly described and presented as a timeline?
3. Was the current clinical condition of the patient on presentation clearly described?
4. Were diagnostic tests or methods and the results clearly described?
5. Was the intervention(s) or treatment procedure(s) clearly described?
6. Was the post-intervention clinical condition clearly described?
7. Were adverse events (harms) or unanticipated events identified and described?
8. Does the case report provide takeaway lessons?

**Appendix 3**: JBI Checklist for Quality of Case Series

1. Were there clear criteria for inclusion in the case series?
2. Was the condition measured in a standard, reliable way for all participants included in the case series?
3. Were valid methods used for the identification of the condition for all participants included in the case series?
4. Did the case series have consecutive inclusion of participants?
5. Did the case series have complete inclusion of participants?
6. Was there transparent reporting of the demographics of the participants in the study?
7. Was there transparent reporting of clinical information of the participants?
8. Were the outcomes of follow-up results of cases reported?
9. Was there transparent reporting of the presenting site(s)/clinic(s) demographic information?
10. Was statistical analysis appropriate?

**Appendix 4**: Jadad Scale for Quality of Randomized Control Trials

1. Was the study designed as randomized?
2. Was the study designed as double-blind?
3. Was there a description of withdrawals and dropouts?
4. Were the objectives of the study defined?
5. Were the outcome measures defined clearly?
6. Was there a clear description of the inclusion and exclusion criteria?
7. Was the sample size justified (for example, power calculation)?
8. Was there a clear description of the interventions?
9. Was there at least one control (comparison) group?
10. Was the method used to assess adverse effects described?
11. Were the methods of statistical analysis described?
