# Supplementary material for: Endoscopic repair of duodenal perforations, a scoping review
Source: Surg Endosc. 2024 Aug 14;38(9):4839–45. doi: 10.1007/s00464-024-11133-x (PMC11362252; doi:10.1007/s00464-024-11133-x)
Supplement: Supplementary file 2 — Supplementary file2 (DOCX 43 KB) [file 464_2024_11133_MOESM2_ESM.docx]

Appendix 5: Case Report Quality Table

| **Authors** | Isaguirre et al^24^ | Ardengh et al^4^ | Huang and Zhu et al^22^ | Ye et al^75^ | Zhen et al^79^ | Li and Han et al^39^ | Wang and Wen et al^68^ | Mao et al^41^ | Li and Ji et al^38^ | Mendoza et al^44^ | Wasserbauer et al^69^ | Meduri et al^43^ | Glatz et al^16^ | Katsinelos et al^29^ |
| --- | --- | --- | --- | --- | --- | --- | --- | --- | --- | --- | --- | --- | --- | --- |
| **Clear Demographics** | No | Yes | Yes | Unclear | Yes | Yes | No | No | Yes | Yes | Unclear | No | Yes | No |
| **Clear History** | No | Yes | No | Unclear | Unclear | Yes | No | No | Yes | Yes | Unclear | No | Yes | Yes |
| **Clear Clinical Presentation** | Yes | Yes | Yes | Yes | Unclear | Yes | Yes | Yes | Yes | Yes | Unclear | No | Yes | Unclear |
| **Clear Diagnostic Tests/ Assessment** | Unclear | Yes | Yes | Yes | Yes | Yes | Yes | Yes | Yes | Yes | Unclear | No | Yes | Unclear |
| **Clear Intervention** | No | Yes | No | Unclear | Unclear | Yes | Yes | Yes | Yes | Yes | Yes | Yes | Yes | Yes |
| **Clear Post Intervention Course** | Unclear | Yes | No | Yes | Unclear | Yes | Yes | No | Yes | No | No | Yes | Yes | Yes |
| **Clear Adverse Events** | No | Yes | No | No | No | Unclear | No | No | Yes | No | No | No | Yes | No |
| **Take Away Lesson Present** | Yes | Yes | Yes | Yes | No | Yes | Yes | Yes | Yes | Yes | Yes | Yes | Yes | Yes |
| **Percent** | 37.5 | 100 | 50 | 68.75 | 50 | 93.75 | 62.5 | 50 | 100 | 75 | 50 | 37.5 | 100 | 62.5 |
| **Study Quality** | Poor | Good | Fair | Fair | Fair | Good | Fair | Fair | Good | Fair | Fair | Poor | Good | Fair |

| **Authors** | Kumar et al^34^ | Sebastian et al^59^ | Mutignani et al^47^ | Anderloni et al^3^ | Mangniovillan et al^40^ | Grande et al^17^ | Takahashi et al^63^ | Kanaya et al^28^ | Miyabe et al^46^ | Watanabe et al^70^ | Furukawa et al^13^ | Tonai et al^66^ | Mishiro et al^45^ |
| --- | --- | --- | --- | --- | --- | --- | --- | --- | --- | --- | --- | --- | --- |
| **Clear Demographics** | Yes | No | No | Yes | Yes | Yes | No | No | No | Yes | Yes | Yes | Yes |
| **Clear History** | Yes | No | No | Yes | Yes | Yes | No | Unclear | No | Yes | Yes | Yes | Yes |
| **Clear Clinical Presentation** | Yes | No | No | Yes | Yes | Yes | Yes | Unclear | Unclear | Yes | Yes | Yes | Yes |
| **Clear Diagnostic Tests/ Assessment** | Yes | Yes | Yes | Yes | Yes | Unclear | Unclear | Yes | Unclear | Yes | Yes | Yes | Yes |
| **Clear Intervention** | Yes | Yes | Yes | Yes | Yes | Yes | Unclear | Yes | Yes | Yes | Yes | Unclear | Yes |
| **Clear Post Intervention Course** | Yes | Unclear | Yes | Yes | No | No | Unclear | Unclear | No | Yes | Yes | Unclear | Yes |
| **Clear Adverse Events** | Yes | No | No | Yes | No | No | No | No | Yes | Yes | Yes | No | Yes |
| **Take Away Lesson Present** | Yes | Yes | Yes | Yes | Yes | Yes | Yes | Yes | Yes | Yes | Yes | Yes | Yes |
| **Percent** | 100 | 43.75 | 50 | 100 | 75 | 68.75 | 43.75 | 56.25 | 50 | 100 | 100 | 75 | 100 |
| **Study Quality** | Good | Poor | Fair | Good | Fair | Fair | Poor | Fair | Fair | Good | Good | Fair | Good |

| **Authors** | Hatogai et al^19^ | Kagawa et al^27^ | Nakagawa et al^48^ | Tanaka et al^64^ | Sugiyama et al^65^ | Nam et al^49^ | Min Lee et al^36^ | Kim,K et al^31^ | Jin Yoo et al^77^ | Han et al^18^ | Holm et al^20^ | Jedrzejczak et al^26^ | Marques de Sa et al^42^ | Leal et al^35^ |
| --- | --- | --- | --- | --- | --- | --- | --- | --- | --- | --- | --- | --- | --- | --- |
| **Clear Demographics** | No | Yes | Yes | Yes | Yes | Yes | Yes | Yes | No | No | Yes | No | No | No |
| **Clear History** | No | No | No | Yes | Yes | Yes | Yes | Yes | No | No | Yes | Yes | Yes | No |
| **Clear Clinical Presentation** | No | Yes | No | Yes | Yes | Yes | Yes | Yes | No | No | Yes | Yes | Yes | Yes |
| **Clear Diagnostic Tests/ Assessment** | No | Yes | No | Yes | Yes | Yes | Yes | Yes | Yes | No | Yes | Yes | Yes | Yes |
| **Clear Intervention** | Yes | Yes | Yes | Yes | Yes | Yes | Yes | Yes | Yes | Yes | Yes | Yes | Yes | Yes |
| **Clear Post Intervention Course** | Yes | Yes | No | No | Yes | Unclear | Yes | Yes | Yes | No | Yes | Yes | Yes | No |
| **Clear Adverse Events** | No | No | No | No | Yes | Unclear | Yes | Yes | No | No | Yes | No | No | No |
| **Take Away Lesson Present** | Yes | Yes | Yes | Yes | Yes | Yes | Yes | Yes | Yes | Yes | Yes | Yes | Yes | Yes |
| **Percent** | 37.5 | 75 | 37.5 | 75 | 100 | 87.5 | 100 | 100 | 50 | 25 | 100 | 75 | 75 | 50 |
| **Study Quality** | Poor | Fair | Poor | Fair | Good | Good | Good | Good | Fair | Poor | Good | Fair | Fair | Fair |

| **Authors** | Bernardo et al^8^ | Yartzev et al^73^ | Yartsev, et al^74^ | Gadzhiev et al^14^ | Yu et al^78^ | Hoon Kim et al^30^ | Choi et al^10^ | Alvaro Martínez-Alcalá García et al^15^ | Salord et al^56^ | Yang and Chen et al^72^ | Rerknimitr et al^55^ | Prachayakul et al^53^ | Dogan et al^11^ |
| --- | --- | --- | --- | --- | --- | --- | --- | --- | --- | --- | --- | --- | --- |
| **Clear Demographics** | No | Yes | Yes | Yes | No | Yes | No | Yes | No | Yes | No | Yes | No |
| **Clear History** | No | Yes | Yes | Yes | No | Yes | Yes | No | No | Yes | No | Yes | Yes |
| **Clear Clinical Presentation** | Yes | Yes | Unclear | Yes | Yes | Yes | Yes | Yes | No | Yes | Yes | No | Unclear |
| **Clear Diagnostic Tests/ Assessment** | Yes | Yes | Yes | Yes | Yes | Yes | Yes | Yes | No | Yes | Unclear | Yes | Yes |
| **Clear Intervention** | Yes | Yes | Yes | Yes | Yes | Yes | Yes | Unclear | Yes | Yes | Yes | Yes | Yes |
| **Clear Post Intervention Course** | No | Yes | Unclear | Yes | Yes | Yes | Yes | Unclear | No | Yes | Unclear | No | Yes |
| **Clear Adverse Events** | Yes | Yes | Yes | Yes | Yes | Yes | Yes | No | No | Yes | Yes | No | No |
| **Take Away Lesson Present** | Yes | Yes | Yes | Yes | Yes | Yes | Yes | Yes | Yes | Yes | Yes | No | Yes |
| **Percent** | 62.5 | 100 | 87.5 | 100 | 75 | 100 | 87.5 | 62.5 | 25 | 100 | 62.5 | 50 | 68.75 |
| **Study Quality** | Fair | Good | Good | Good | Fair | Good | Good | Fair | Poor | Good | Fair | Fair | Fair |

| **Authors** | Abbitt et al^1^ | Ahlawat et al^2^ | Sanders et al^58^ | Lee et al^37^ | Seibert et al^60^ | Baron et al^7^ | Solomon et al^61^ | Yoo et al^76^ | Ferm et al^12^ | Kirtane et al^32^ | Kriss et al^33^ | Samarasena et al^57^ | Matsuoka et al^80^ |
| --- | --- | --- | --- | --- | --- | --- | --- | --- | --- | --- | --- | --- | --- |
| **Clear Demographics** | No | No | No | No | Yes | No | No | Yes | Yes | No | No | Yes | Yes |
| **Clear History** | No | No | No | Unclear | No | Unclear | No | Yes | Yes | Yes | Yes | Yes | Yes |
| **Clear Clinical Presentation** | Unclear | Yes | Yes | Unclear | Yes | Unclear | Yes | Yes | Yes | No | No | Yes | Yes |
| **Clear Diagnostic Tests/Assessment** | Yes | Yes | Yes | Unclear | Unclear | Yes | Unclear | Yes | Yes | Yes | No | Yes | Yes |
| **Clear Intervention** | Yes | Yes | Yes | Yes | Yes | Yes | Yes | Yes | Yes | Unclear | No | Yes | Yes |
| **Clear Post Intervention Course** | No | Yes | Yes | No | Unclear | Yes | Unclear | Yes | Yes | No | No | Yes | Yes |
| **Clear Adverse Events** | No | Yes | No | No | No | Yes | Yes | No | Yes | No | No | Yes | Yes |
| **Take Away Lesson Present** | Yes | Yes | Yes | No | Yes | Yes | Yes | Yes | Yes | Yes | No | Yes | Yes |
| **Percent** | 43.75 | 75 | 62.5 | 31.25 | 62.5 | 75 | 62.5 | 87.5 | 100 | 43.75 | 12.5 | 100 | 100 |
| **Study Quality** | Poor | Fair | Fair | Poor | Fair | Fair | Fair | Good | Good | Poor | Poor | Good | Good |

| **Authors** | Barakat et al^6^ | Voellinger et al^67^ | Issak et al^25^ | Bansal et al^5^ | Rau et al^54^ | Noorani et al^51^ | Honig et al^21^ | Natov et al^50^ | Wu et al^71^ | Charabaty-Pishvaian et al^9^ | Ince et al^23^ | Ohara et al^52^ |
| --- | --- | --- | --- | --- | --- | --- | --- | --- | --- | --- | --- | --- |
| **Clear Demographics** | Yes | No | No | Yes | No | Yes | Yes | Unclear | No | No | No | Yes |
| **Clear History** | Yes | Yes | No | Yes | No | Yes | Yes | Yes | Yes | No | No | Yes |
| **Clear Clinical Presentation** | Yes | No | Yes | Yes | Yes | No | Yes | Yes | Yes | No | Yes | Yes |
| **Clear Diagnostic Tests/ Assessment** | Yes | Yes | Yes | Yes | Yes | Yes | Yes | Yes | Yes | Yes | Yes | Yes |
| **Clear Intervention** | Yes | No | Yes | Yes | Yes | Yes | Yes | Yes | Yes | Yes | Yes | Yes |
| **Clear Post Intervention Course** | Yes | No | Yes | No | No | No | Yes | No | No | Yes | Yes | Yes |
| **Clear Adverse Events** | Yes | No | Yes | No | Yes | na | Yes | not applicable | Yes | Yes | Yes | Yes |
| **Take Away Lesson Present** | Yes | Yes | Yes | Yes | Yes | Yes | Yes | No | Yes | Yes | Yes | Yes |
| **Percent** | 100 | 37.5 | 75 | 75 | 62.5 | 62.5 | 100 | 56.25 | 75 | 62.5 | 75 | 100 |
| **Study Quality** | Good | Poor | Fair | Fair | Fair | Fair | Good | Fair | Fair | Fair | Fair | Good |

References:

1. Abbitt, D., et al. (2021). "Endoluminal vacuum closure of a duodenal perforation." Journal of Surgical Case Reports 2021(11): rjab479.

2. Ahlawat, S. K. and N. Haddad (2009). "Repair of an EUS--induced duodenal perforation with endoscopic clips." Acta gastro-enterologica Belgica 72(3): 361-364.

3. Anderloni, A., et al. (2017). "Successful endoscopic closure of iatrogenic duodenal perforation with the new Padlock Clip." Endoscopy 49(S 01): E58-E59.

4. Ardengh, J. C., et al. (2020). "Successful closure of a complicated endoscopic ultrasound-related duodenal perforation." Endoscopy 52(02): E53-E54.

5. Bansal, R., et al. (2018). "A case of unresolved and worsening retroperitoneal abscess." Case Reports in Gastrointestinal Medicine 2018.

6. Barakat, M. T., et al. (2018). "Cut and paste: endoscopic management of a perforating biliary stent utilizing scissors and clips." Digestive diseases and sciences 63: 2202-2205.

7. Baron, T. H., et al. (2000). "Hemoclip repair of a sphincterotomy-induced duodenal perforation." Gastrointestinal Endoscopy 52(4): 566-568.

8. Bernardo, S., et al. (2020). "Endoscopic closure of a duodenal perforation caused by early migration of a biliary plastic stent with an over-the-scope-clip." Gastroenterologia y Hepatologia 44(2): 139-140.

9. Charabaty-Pishvaian, A. and F. Al-Kawas (2004). "Endoscopic treatment of duodenal perforation using a clipping device: case report and review of the literature." Southern medical journal 97(2): 190-194.

10. Choi, S. H., et al. (2018). "Liver abscess secondary to perforation after duodenal endoscopic resection." The Korean Journal of Gastroenterology 71(5): 286-289.

11. Dogan, U., et al. (2013). "Endoscopic closure of an endoscope-related duodenal perforation using the over-the-scope clip." TURKISH JOURNAL OF GASTROENTEROLOGY 24(5).

12. Ferm, S., et al. (2018). "Primary endoscopic closure of duodenal perforation secondary to biliary stent migration: a case report and review of the literature." Journal of Investigative Medicine High Impact Case Reports 6: 2324709618792031.

13. Furukawa, K., et al. (2016). "Endoscopic Closure of Duodenal Perforation with the Over-the-scope-clipping System." Internal Medicine 55(21): 3131-3135.

14. Gadzhiev, N., et al. (2018). "Novel approach for endoscopic management of duodenal injury during perirenal infected haematoma drainage after shock-wave lithotripsy." Case Reports in Urology 2018.

15. García, A. M.-A., et al. (2016). "Large type I post-ERCP perforation closed immediately through the duodenoscope with through-the-scope endoclips." Endoscopy 48(S 01): E86-E87.

16. Glatz, T., et al. (2015). "Vacuum sponge therapy using the pull-through technique via a percutaneous endoscopic gastrostomy to treat iatrogenic duodenal perforation." Endoscopy 47(S 01): E567-E568.

17. Grande, G., et al. (2016). "Quick, safe and effective repair of EUS-related duodenal perforation using over-the-scope clip system (with video)." Digestive and Liver Disease 48(9): 1099-1100.

18. Han, S., et al. (2017). "Endoscopic Management of Gastrointestinal Leaks and Perforation with Polyglycolic Acid Sheets." ce 50(3): 293-296.

19. Hatogai, K., et al. (2013). "Unexpected endoscopic full-thickness resection of a duodenal neuroendocrine tumor." World J Gastroenterol 19(26): 4267-4270.

20. Holm, T. E., et al. (2019). "Endoscopic stent treatment of a duodenal ulcer perforation using a semi‐covered stent." Clinical Case Reports 7(8): 1554-1556.

21. Honig, S. E., et al. (2020). "Advanced endoscopic rescue of a complication (duodenojejunostomy leak) after a pylorus-preserving pancreaticoduodenectomy in a post-esophagectomy patient with pancreatic adenocarcinoma: a case report and review of the literature." Journal of Pancreatic Cancer 6(1): 5-11.

22. Huang, S. and S. Zhu (2017). "Closure of duodenal ulcer perforation using a novel endoloop device with a single-channel gastroscope after failed laparoscopic repair." Endoscopy 49(S 01): E31-E32.

23. İnce, A. T., et al. (2014). "Endoscopic repair of duodenal perforation with over-the-scope clipping system and endoclips: A case report." Turk J Gastroenterol 25(1): 103-105.

24. Isaguirre, J., et al. (2008). "Endoscopic treatment of duodenal perforation following laparoscopic cholecystectomy." Endoscopy 40(S 02): E138-E138.

25. Issak, A. and M. Musleh (2020). "Successful closure of chronic recurrent Enterocutaneous fistula with a concurrent over‐the‐scope closure and a stent placement." Clinical Case Reports 8(2): 239-242.

26. Jędrzejczak, B., et al. (2020). "Endoscopic management of duodenal fistula in a patient operated after abdominal injury." Polish Journal of Surgery 92(2): 64-67.

27. Kagawa, T. J., Atsushi; Yoshida, Keigo; Miyagi, Takuya (2019). "SUCCESSFUL ENDOSCOPIC MANAGEMENT OF DUODENAL PERFORATION THAT OCCURRED DURING ENDOSCOPIC RETROGRADE CHOLANGIOPANCREATOGRAPHY." Gastroenterological Endoscopy 61(10): 2371.

28. Kanaya, K., et al. (2021). "Endoscopic clipping to prevent papillary obstruction when closing a duodenal perforation with an over-the-scope clip." VideoGIE 6(6): 266-268.

29. Katsinelos, P., et al. (2005). "Treatment of a duodenal perforation secondary to an endoscopic sphincterotomy with clips." World J Gastroenterol 11(39): 6232-6234.

30. Kim, D. H., et al. (2019). "Endoluminal closure of an unrecognized penetrating stab wound of the duodenum with endoscopic band ligation: A case report." World Journal of Clinical Cases 7(20): 3271.

31. Kim, K., et al. (2016). "Repair of an Endoscopic Retrograde Cholangiopancreatography-Related Large Duodenal Perforation Using Double Endoscopic Band Ligation and Endoclipping." Clinical Endoscopy 50(2): 202-205.

32. Kirtane, T. and S. Singhal (2016). "Endoscopic closure of iatrogenic duodenal perforation using dual over-the-scope clips." Gastrointestinal Endoscopy 83(2): 467-468.

33. Kriss, M., et al. (2015). "Duodenal perforation secondary to migrated biliary stent in a liver transplant patient: successful endoscopic closure with an over-the-scope clip." Gastrointestinal Endoscopy 81(5): 1258-1259.

34. Kumar, N., et al. (2022). "Endoscopic Closure of Large Iatrogenic Duodenal Perforation: Right Use of Endoscopic Accessories." Journal of Digestive Endoscopy 13(02): 113-115.

35. Leal, T., et al. (2021). "Endoscopic resolution of a duodenal perforation due to a pancreatic stent." Rev Esp Enferm Dig 113(5): 383.

36. Lee, J. M. and C. B. Rim (2021). "Endoscopic Band Ligation in Endoscopic Retrograde Cholangiopancreatography Related Duodenal Perforation." Korean Journal Gastroenterology 77(3): 136-140.

37. Lee, T. H., et al. (2008). "Endoscopic management of duodenal perforation secondary to ingestion of an uncommon foreign body." Gastrointestinal Endoscopy 67(4): 729-731.

38. Li, Q., et al. (2015). "ERCP-induced duodenal perforation successfully treated with endoscopic purse-string suture: a case report." Oncotarget 6(19): 17847-17850.

39. Li, Y., et al. (2014). "Successful Closure of Lateral Duodenal Perforation by Endoscopic Band Ligation After Endoscopic Clipping Failure." Official journal of the American College of Gastroenterology | ACG 109(2): 293-295.

40. Mangiavillano, B., et al. (2014). "Successful closure of an endoscopic ultrasound-induced duodenal perforation using an over-the-scope-clip." Endoscopy 46(S 01): E206-E207.

41. Mao, X., et al. (2020). "Imaging findings and clinical features of atypical retroperitoneal abscess caused by duodenal perforation: a case report and review of the literature." Journal of Medical Case Reports 14(1): 105.

42. Marques de Sá, I., et al. (2021). "Biliary Stent Migration With Duodenal Perforation–How to Manage?" Endoscopy 53: S215.

43. Meduri, B., et al. (2014). "Endoscopic ultrasound-guided fine needle aspiration and endoscopic biliary drainage following closure of a duodenal perforation with an over-the-scope clip." Endoscopy 46(S 01): E69-E70.

44. Mendoza, J., et al. (2021). "Hepatoduodenal fistula closure diagnosed and characterized Ecoendoscopically (EUS) and managed by OTSC CLIP OVESCO: A case report." International Journal of Surgery Case Reports 84: 106093.

45. Mishiro, T., et al. (2016). "Successful Endoscopic Management of Non-Healing Perforated Duodenal Ulcer with Polyglycolic Acid Sheet and Fibrin Glue." ACG Case Rep J 3(4): e197.

46. Miyabe, K., et al. (2021). "Over-the-scope-clip treatment for perforation of the duodenum after endoscopic papillectomy." VideoGIE 6(2): 101-104.

47. Mutignani, M., et al. (2006). "Successful endoscopic closure of a lateral duodenal perforation at ERCP with fibrin glue." Gastrointestinal Endoscopy 63(4): 725-727.

48. Nakagawa, Y., et al. (2010). "Endoscopic closure of a large ERCP-related lateral duodenal perforation by using endoloops and endoclips." Gastrointestinal Endoscopy 72(1): 216-217.

49. Nam, H. S., et al. (2011). "A Case of Duodenal Perforation Caused by Biliary Plastic Stent Treated with Approximation using Endoclip and Detachable Snare." Korean Journal Gastroenterology 57(2): 129-133.

50. Natov, P. S., et al. (2019). "2167 Endoscopic Closure of a Duodeno-Pleural Fistula Secondary to Transarterial Chemoembolization." Official journal of the American College of Gastroenterology| ACG 114: S1210-S1211.

51. Noorani, S. and F. Rashti (2020). A Choledochoduodenal Fistula Masquerading as an Upper GI Bleed. AMERICAN JOURNAL OF GASTROENTEROLOGY, LIPPINCOTT WILLIAMS & WILKINS TWO COMMERCE SQ, 2001 MARKET ST, PHILADELPHIA ….

52. Ohara, Y., et al. (2017). "Enormous postoperative perforation after endoscopic submucosal dissection for duodenal cancer successfully treated with filling and shielding by polyglycolic acid sheets with fibrin glue and computed tomography-guided abscess puncture." Clinical Journal of Gastroenterology 10(6): 524-529.

53. Prachayakul, V., et al. (2012). "Duodenal perforation due to plastic stent migration successfully treated by endoscopy." Gastrointestinal Endoscopy 75(6): 1265-1266.

54. Rau, P., et al. (2022). "Use of a novel helical tack system for the management of challenging upper gastrointestinal defects." VideoGIE 7(2): 85-88.

55. Rerknimitr, R., et al. (2008). "Use of endoclips to close sphincterotomy-related perforation." Endoscopy 40(S 02): E169-E169.

56. Salord, S., et al. (2012). "Endoscopic closure of duodenal perforation with an over-the-scope clip during endoscopic ultrasound-guided cholangiopancreatography." Revista Española de Enfermedades Digestivas, 2012, vol. 104, num. 9, p. 489-490.

57. Samarasena, J., et al. (2012). "Endoscopic closure of an iatrogenic duodenal perforation: a novel technique using endoclips, endoloop, and fibrin glue." Endoscopy 44(S 02): E424-e425.

58. Sanders, M. K., et al. (2008). "Endoscopic closure of iatrogenic duodenal perforation during EUS in a patient with unusual anatomy." Gastrointestinal Endoscopy 68(4): 802-804.

59. Sebastian, S., et al. (2004). "Endoscopic Closure of Iatrogenic Duodenal Perforation during Endoscopic Ultrasound." Endoscopy 36(03): 245-245.

60. Seibert, D. (2003). "Use of an endoscopic clipping device to repair a duodenal perforation." Endoscopy 35(02): 189-189.

61. Solomon, M., et al. (2012). "Iatrogenic duodenal perforation treated with endoscopic placement of metallic clips: a case report." Case reports in Medicine 2012.

62. Stavrou, G., et al. (2019). "Successful closure of a complicated duodenal ulcer perforation with an expandable esophageal stent." Asian Journal of Endoscopic Surgery 12(3): 326-328.

63. Takahashi, K., et al. (2021). "Successful endoscopic closure with endoscopic clips for endoscopic ultrasound related large duodenal perforation." Journal of Rural Medicine 16(3): 165-169.

64. Tanaka, Y., et al. (2016). Successful treatment of a perforated duodenal ulcer using polyglycolic acid sheets. Journal of Gastroenterology and Hepatology, WILEY-BLACKWELL 111 RIVER ST, HOBOKEN 07030-5774, NJ USA.

65. Tomohiko Sugiyama, R. T., Tomio Ogiso, Satoko Tajirishita, Mitsuru Okuno, Chiemi Nakayama, Eisuke Ogiso, Akihiko Sugiyama, Norihiro Kato, Eiichi Tomita (2021). "A case in which endoscopic treatment using an Over-The-Scope Clip (OTSC) was effective for duodenal perforation due to accidental ingestion of PTP." Journal of the Japanese Gastrointestinal Endoscopy Society 63(1): 38-44.

66. Tonai, Y., et al. (2016). "Iatrogenic duodenal perforation during underwater ampullectomy: endoscopic repair using polyglycolic acid sheets." Endoscopy 48(S 01): E97-E98.

67. Voellinger, M. T., et al. (2014). "Enterocutaneous fistula from a billroth II afferent limb: successful closure with endoclips." ACG Case Reports Journal 1(2): 76.

68. Wang, L., et al. (2016). "Endoscopic Removal of a Duodenal-Perforating Leg of Glasses with Dormia Basket." Case Reports in Gastroenterology 10(3): 679-684.

69. Wasserbauer, M. L., J; Keil, R (2014). "OVESCO clip as a solution of an ERCP complication." Gastroent Hepatol 68(6): 485-487.

70. Watanabe, K., et al. (2018). "Successful Endoscopic Closure Using Polyglycolic Acid Sheets with Fibrin Glue for Nonhealing Duodenal Ulcer with Perforation after Proton Beam Therapy of Liver Tumor." Case Reports in Gastroenterology 12(3): 679-685.

71. Wu, P.-H., et al. (2019). "Successful endoscopic management of double iatrogenic perforations induced by endoscopic retrograde cholangiopancreatography and computed tomography-guided colon drainage." Case Reports in Gastroenterology 13(1): 1-5.

72. Yang, H.-Y. and J.-H. Chen (2015). "Endoscopic fibrin sealant closure of duodenal perforation after endoscopic retrograde cholangiopancreatography." World journal of gastroenterology 21(45): 12976.

73. Yartsev, P., et al. (2020). "Endoscopic treatment of perforated duodenal ulcer using a partially polyurethane‐covered self‐expandable nitinol stent: A case report." Asian Journal of Endoscopic Surgery 13(1): 103-106.

74. Yartsev, P., et al. (2020). "Endoscopic treatment of gastroduodenal perforations." Khirurgiia(4): 61-64.

75. Ye, L., et al. (2019). "Endoscopic partial closure followed by adequate drainage for treating delayed perforation caused by duodenal endoscopic submucosal dissection: A case report." Medicine (Baltimore) 98(22): e15883.

76. Yoo, T., et al. (2018). "Successful repair of duodenal perforation with endoscopic vacuum therapy." Gastrointestinal Endoscopy 87(5): 1363-1364.

77. Yoo, Y. J., et al. (2018). "Covered Self-expandable Metallic Stent Insertion as a Rescue Procedure for Postoperative Leakage after Primary Repair of Perforated Duodenal Ulcer." kjg 72(5): 262-266.

78. Yu, D. W., et al. (2014). "Endoscopic treatment of duodenal fistula after incomplete closure of ERCP-related duodenal perforation." World Journal of Gastrointestinal Endoscopy 6(6): 260.

79. Zeng, C.-Y., et al. (2014). "Single-channel endoscopic closure of ERCP-related large duodenal perforations." Endoscopy 46(S 01): E603-E604.

80. Matsuoka, M, et al. (2021). “Endoscopic Closure of an Acute Duodenal Perforation Occurring during Endoscopic Ultrasound Using Endoclips and Polyglycolic Acid Sheets with Fibrin Glue.” Case Rep Gastroenterol 15 (1): 253–261.
