# Supplementary material for: Endoscopic repair of duodenal perforations, a scoping review
Source: Surg Endosc. 2024 Aug 14;38(9):4839–45. doi: 10.1007/s00464-024-11133-x (PMC11362252; doi:10.1007/s00464-024-11133-x)
Supplement: Supplementary file 3 — Supplementary file3 (DOCX 46 KB) [file 464_2024_11133_MOESM3_ESM.docx]

Appendix 6: Case Series Quality Table

| **Authors** | Klein et al^31^ | Bureau et al^6^ | Wang and Zhou et al^65^ | Wang and Qu et al^64^ | Zhu et al^70^ | Liu et al^37^ | Li and Dou et al^36^ | Zeng et al^69^ | Li and Ji et al^34^ | Li and Yan et al^35^ | Donatelli and Vergeau et al^13^ | Amor et al^2^ | Donatelli and Cereatti et al^12^ |
| --- | --- | --- | --- | --- | --- | --- | --- | --- | --- | --- | --- | --- | --- |
| **Inclusion Criteria** | Yes | Yes | Yes | Yes | Yes | Yes | No | Yes | Yes | Yes | Yes | Yes | Yes |
| **Standard Measurement** | Yes | Yes | Yes | Yes | Yes | Yes | No | No | No | Yes | Yes | Yes | Yes |
| **Valid Methods** | Yes | Yes | Yes | Yes | Yes | Yes | No | Yes | Yes | Yes | Yes | Yes | Yes |
| **Consecutive Participants** | Yes | Yes | Yes | Yes | Yes | Yes | Yes | No | Yes | Yes | Yes | Unclear | Yes |
| **Complete Inclusion** | Yes | Yes | Yes | Yes | Yes | Yes | Yes | Yes | Yes | Yes | Yes | Unclear | Yes |
| **Clear Demographics** | Yes | No | Yes | Yes | Yes | Yes | No | No | Yes | Yes | No | No | Yes |
| **Clear Clinical Information** | Yes | Yes | Yes | Yes | Yes | Yes | No | No | No | Yes | Yes | Yes | Yes |
| **Clear Outcomes** | Yes | Yes | Yes | Yes | Yes | Yes | Yes | Yes | No | No | Yes | Yes | Yes |
| **Clear Presentation** | Yes | Yes | Yes | No | Yes | No | No | No | No | Yes | No | No | Yes |
| **Appropriate Statistical Analysis** | Yes | Yes | Yes | n/a | Yes | n/a | Yes | n/a | Yes | Yes | n/a | n/a | Yes |
| **Percent** | 100 | 90 | 100 | 89 | 100 | 89 | 40 | 4 | 60 | 90 | 78 | 67 | 100 |
| **Study Quality** | Good | Good | Good | Good | Good | Good | Poor | Poor | Fair | Good | Fair | Fair | Good |

| **Authors** | Wedi et al^66^ | Khater et al^27^ | El bacha et al^14^ | Wichmann et al^67^ | Fischer et al^15^ | Hagel et al^17^ | Raithel et al^52^ | Loske et al^38^ | Decassian et al^9^ | Theopistos et al^60^ | Goenka et al^16^ | Dahale et al^8^ |
| --- | --- | --- | --- | --- | --- | --- | --- | --- | --- | --- | --- | --- |
| **Inclusion Criteria** | Yes | Yes | Yes | Yes | Yes | Yes | Yes | Yes | Yes | Yes | Yes | Yes |
| **Standard Measurement** | Yes | No | Yes | Yes | Yes | Yes | Yes | No | Yes | Yes | Yes | Yes |
| **Valid Methods** | Yes | Yes | Yes | Yes | Yes | Yes | Yes | Yes | Yes | Yes | Yes | Yes |
| **Consecutive Participants** | Yes | Yes | Yes | Unclear | Unclear | Yes | Unclear | No | Yes | Yes | Unclear | Yes |
| **Complete Inclusion** | Yes | Yes | Yes | Unclear | Unclear | Yes | Yes | No | Yes | Yes | Unclear | Yes |
| **Clear Demographics** | Yes | No | Yes | Yes | No | Yes | No | Yes | No | Yes | Yes | Yes |
| **Clear Clinical Information** | Yes | No | Yes | Yes | Yes | Yes | No | Yes | No | Yes | No | Yes |
| **Clear Outcomes** | Yes | Yes | Yes | Yes | Yes | Yes | Unclear | Yes | No | Yes | No | Yes |
| **Clear Presentation** | No | No | Yes | No | No | Yes | No | Yes | No | Yes | No | Yes |
| **Appropriate Statistical Analysis** | Yes | Yes | Yes | n/a | n/a | Yes | Yes | n/a | Yes | n/a | n/a | Yes |
| **Percent** | 90 | 60 | 100 | 78 | 67 | 100 | 60 | 67 | 60 | 100 | 56 | 100 |
| **Study Quality** | Good | Fair | Good | Fair | Fair | Good | Fair | Fair | Fair | Good | Fair | Good |

| **Authors** | Joshi et al^24^ | Nasa and Choudhary et al^42^ | Nasa and Sharma et al^43^ | Parodi et al^51^ | Tringali et al^61^ | Amodio et al^1^ | Dinelli et al^10^ | Mangiavillano et al^39^ | Sbrozzi-Vanni et al^54^ | Kessoku et al^26^ | Iwasa et al^20^ | Nishiyama et al^45^ |
| --- | --- | --- | --- | --- | --- | --- | --- | --- | --- | --- | --- | --- |
| **Inclusion Criteria** | Yes | No | No | Yes | Yes | Yes | Yes | Yes | Yes | Yes | Yes | Yes |
| **Standard Measurement** | Yes | Yes | No | Yes | Yes | Yes | Unclear | Yes | Yes | Yes | Yes | Yes |
| **Valid Methods** | Yes | No | No | Unclear | Yes | Yes | Yes | Yes | Yes | Yes | Yes | Unclear |
| **Consecutive Participants** | No | Unclear | Yes | Yes | Yes | No | Yes | Yes | Yes | Yes | Yes | Yes |
| **Complete Inclusion** | Unclear | Unclear | Yes | Yes | Yes | No | Yes | Yes | Yes | Yes | Yes | Yes |
| **Clear Demographics** | Yes | No | No | No | Yes | No | Yes | No | Yes | Yes | Yes | Yes |
| **Clear Clinical Information** | Yes | No | No | No | Yes | Yes | No | No | Yes | Yes | Yes | Yes |
| **Clear Outcomes** | Yes | No | No | Unclear | Yes | Yes | Yes | No | Yes | Yes | Yes | Yes |
| **Clear Presentation** | Yes | No | No | No | Yes | No | No | No | Yes | No | Yes | Yes |
| **Appropriate Statistical Analysis** | n/a | Yes | Yes | Yes | Yes | n/a | Yes | Yes | Yes | n/a | Yes | n/a |
| **Percent** | 83 | 30 | 30 | 60 | 100 | 56 | 75 | 60 | 100 | 89 | 100 | 94 |
| **Study Quality** | Good | Poor | Poor | Fair | Good | Fair | Fair | Fair | Good | Good | Good | Good |

| **Authors** | Takimoto et al^57^ | Miura et al^40^ | Kaneko et al^25^ | Yahagi et al^68^ | Ojima et al^47^ | Dohi et al^11^ | Choi et al^7^ | Jin and Jeong et al^23^ | Lee and Bang et al^33^ | Seo et al^55^ | Jeon et al^22^ | Voermans et al^63^ |
| --- | --- | --- | --- | --- | --- | --- | --- | --- | --- | --- | --- | --- |
| **Inclusion Criteria** | Yes | Yes | Yes | Yes | Yes | Yes | Yes | No | Yes | Yes | Yes | Yes |
| **Standard Measurement** | Yes | Yes | Yes | Yes | Yes | Yes | Yes | Unclear | Yes | No | Yes | Yes |
| **Valid Methods** | Yes | Unclear | Yes | Yes | Yes | Yes | Yes | Unclear | Yes | No | Yes | Yes |
| **Consecutive Participants** | Yes | Unclear | No | Yes | Yes | No | Unclear | No | Yes | Yes | Yes | Yes |
| **Complete Inclusion** | Yes | Yes | No | Yes | Yes | No | Unclear | Yes | Yes | Yes | Yes | Yes |
| **Clear Demographics** | Yes | Yes | Unclear | Yes | Yes | Yes | No | No | Yes | No | Unclear | Yes |
| **Clear Clinical Information** | Yes | Yes | Yes | Yes | Yes | No | Yes | No | Yes | No | Unclear | Yes |
| **Clear Outcomes** | Yes | Yes | Yes | Yes | Yes | Yes | Yes | Yes | Yes | Yes | Unclear | Yes |
| **Clear Presentation** | Yes | Yes | Unclear | Yes | Yes | No | No | Yes | Yes | No | Unclear | Yes |
| **Appropriate Statistical Analysis** | Yes | Yes | Yes | Yes | Yes | Yes | n/a | Yes | Yes | Yes | Yes | Yes |
| **Percent** | 100 | 90 | 70 | 100 | 100 | 60 | 67 | 50 | 100 | 50 | 80 | 100 |
| **Study Quality** | Good | Good | Fair | Good | Good | Fair | Fair | Fair | Good | Fair | Good | Good |

| **Authors** | Stassen et al^56^ | Santos et al^53^ | Nedoluzhko et al^44^ | Kim and Kim et al^29^ | Kim and Moon et al^30^ | Park and Ham et al^48^ | Bergström et al^4^ | Valli et al^62^ | Honegger et al^19^ | Angsuwatcharakon et al^3^ | Parlak and Koksal et al^50^ |
| --- | --- | --- | --- | --- | --- | --- | --- | --- | --- | --- | --- |
| **Inclusion Criteria** | Yes | Yes | Yes | Yes | Yes | Yes | Yes | Yes | Yes | Yes | Yes |
| **Standard Measurement** | Yes | Yes | Yes | Yes | Yes | Yes | Yes | Yes | Yes | Yes | Yes |
| **Valid Methods** | Yes | Yes | Yes | Yes | Yes | Yes | Yes | Yes | Yes | Yes | Yes |
| **Consecutive Participants** | Yes | Yes | Yes | Yes | Yes | No | Yes | Yes | Unclear | Yes | Yes |
| **Complete Inclusion** | Yes | Yes | Yes | Yes | Yes | No | Yes | Yes | Unclear | Yes | Yes |
| **Clear Demographics** | Unclear | Yes | No | No | Yes | No | Yes | Yes | Unclear | Yes | Yes |
| **Clear Clinical Information** | Yes | Yes | No | Yes | Yes | No | Yes | Yes | Yes | Yes | Yes |
| **Clear Outcomes** | Yes | Yes | Yes | Yes | Yes | Yes | Yes | Yes | Yes | Yes | Yes |
| **Clear Presentation** | Yes | Yes | No | No | Yes | No | Yes | Yes | No | Yes | Yes |
| **Appropriate Statistical Analysis** | Yes | Yes | Yes | Yes | Yes | Yes | Yes | Yes | Yes | Yes | Yes |
| **Percent** | 95 | 100 | 70 | 80 | 100 | 50 | 100 | 100 | 75 | 100 | 100 |
| **Study Quality** | Good | Good | Fair | Good | Good | Fair | Good | Good | Fair | Good | Good |

| **Authors** | Parlak, Koksal and Disibeyaz et al^49^ | Odemis et al^46^ | Tavusbay et al^58^ | Kocataş et al^32^ | Khokhar et al^28^ | Thapa et al^59^ | Haito-Chavez et al^18^ | Bill et al^5^ | Moyer et al^41^ | Jamil et al^21^ | Hoibian et al^71^ |
| --- | --- | --- | --- | --- | --- | --- | --- | --- | --- | --- | --- |
| **Inclusion Criteria** | Yes | Yes | Yes | Yes | Yes | Yes | Yes | Yes | No | No | Yes |
| **Standard Measurement** | Yes | Yes | Yes | No | Yes | Yes | Yes | Yes | Yes | Yes | Yes |
| **Valid Methods** | Yes | Yes | Yes | No | Yes | Yes | Yes | Yes | Yes | Yes | Yes |
| **Consecutive Participants** | Yes | Yes | Yes | No | Unclear | Yes | Yes | Yes | No | Yes | Yes |
| **Complete Inclusion** | Yes | Yes | Yes | No | Unclear | Yes | Unclear | Yes | No | Yes | Yes |
| **Clear Demographics** | Yes | Yes | No | Yes | Yes | Yes | No | Yes | No | No | No |
| **Clear Clinical Information** | Yes | Yes | No | Yes | Yes | Yes | Yes | Yes | Unclear | No | No |
| **Clear Outcomes** | Yes | Yes | No | Yes | Yes | Yes | Unclear | Yes | Yes | Yes | No |
| **Clear Presentation** | No | Yes | No | No | No | Yes | No | Yes | No | No | No |
| **Appropriate Statistical Analysis** | n/a | Yes | Yes | Yes | n/a | Yes | Yes | Yes | Yes | Yes | Yes |
| **Percent** | 89 | 100 | 60 | 50 | 78 | 100 | 70 | 100 | 45 | 60 | 60 |
| **Study Quality** | Good | Good | Fair | Fair | Fair | Good | Fair | Good | Poor | Fair | Fair |

**References**:

1. Amodio, P. M., et al. (2007). "Selected treatments for duodenal perforation after ERCP. A report of three cases." Chirurgia Italiana 59(3): 343-346.

2. Amor, W. H., et al. (2012). "Successful endoscopic management of large upper gastrointestinal perforations following EMR using over-the-scope clipping combined with stenting." Endoscopy 44(S 02): E277-E278.

3. Angsuwatcharakon, P., et al. (2016). "Efficacy of the Ovesco clip for closure of endoscope related perforations." Diagnostic and Therapeutic Endoscopy 2016.

4. Bergström, M., et al. (2012). "Self-expandable metal stents as a new treatment option for perforated duodenal ulcer." Endoscopy: 222-225.

5. Bill, J. G., et al. (2018). "The importance of early recognition in management of ERCP-related perforations." Surgical Endoscopy 32: 4841-4849.

6. Bureau, M.-A., et al. (2020). "Lateral duodenal wall perforation due to plastic biliary stent migration: a case series of endoscopic closure." Endoscopy International Open 8(05): E573-E577.

7. Choi, H. J., et al. (2013). "The temporary placement of covered self-expandable metal stents to seal various gastrointestinal leaks after surgery." Gut and Liver 7(1): 112.

8. Dahale, A. S., et al. (2021). "Management of scope-induced type I duodenal perforations: over-the-scope clip versus surgery." Indian Journal of Gastroenterology 40(3): 287-294.

9. Decassian, T. and M. Dauer (2019). "APP–Das Amberger Perforationsprojekt als Grundlage für die Etablierung eines Komplikationserfassungs-und Managementsystems (KEMS) in der Endoskopie." Zeitschrift für Gastroenterologie 57(08): 960-970.

10. Dinelli, M., et al. (2017). "First clinical experiences with a novel endoscopic over-the-scope clip system." Endoscopy International Open 5(03): E151-E156.

11. Dohi, O., et al. (2020). "Efficacy and safety of endoscopic submucosal dissection using a scissors‐type knife with prophylactic over‐the‐scope clip closure for superficial non‐ampullary duodenal epithelial tumors." Digestive Endoscopy 32(6): 904-913.

12. Donatelli, G., et al. (2013). "Closure with an over-the-scope clip allows therapeutic ERCP to be safely performed after acute duodenal perforation during diagnostic endoscopic ultrasound." Endoscopy 45(S 02): E392-E393.

13. Donatelli, G. V., B.M.; Dritsas, S.; Dumont, J.-L.; Tuszynski, T.; Meduri, B.; (2013). "Closure with an over-the-scope clip allows therapeutic ERCP to be safely performed after acute duodenal perforation during diagnostic endoscopic ultrasound." Endoscopy 45(0): 392-393.

14. El Bacha, H. P., H (2019). "Over the scope clips for EUS duodenal perforation." United European Gastroenterology Journal 7(8): 853-854.

15. Fischer, A., et al. (2016). "Two-sided sponge (TSS) treatment: Description of a novel device and technique for endoscopic vacuum treatment (EVT) in the upper gastrointestinal tract." Endoscopy International Open 4(09): E937-E940.

16. Goenka, M. K., et al. (2017). "Endoscopic management of gastrointestinal leaks and bleeding with the over-the-scope clip: a prospective study." Clinical Endoscopy 50(1): 58-63.

17. Hagel, A. F., et al. (2012). "Over-the-scope clip application yields a high rate of closure in gastrointestinal perforations and may reduce emergency surgery." Journal of Gastrointestinal Surgery 16: 2132-2138.

18. Haito-Chavez, Y., et al. (2014). "International multicenter experience with an over-the-scope clipping device for endoscopic management of GI defects (with video)." Gastrointestinal Endoscopy 80(4): 610-622.

19. Honegger, C., et al. (2017). "Establishment of Over-The-Scope-Clips (OTSC®) in daily endoscopic routine." United European Gastroenterology Journal 5(2): 247-254.

20. Iwasa, Y., et al. (2020). "The efficacy of over-the-scope clip closure for gastrointestinal iatrogenic perforation during endoscopic ultrasound and endoscopic retrograde cholangiopancreatography for pancreaticobiliary diseases." Surgical Laparoscopy, Endoscopy & Percutaneous Techniques 30(3): 257-262.

21. Jamil, L. H., et al. (2017). "Safety and efficacy of cap-assisted EMR for sporadic nonampullary duodenal adenomas." Gastrointestinal Endoscopy 86(4): 666-672.

22. Jeon, J. K., T.; Ryu, Y. (2015). "Management of endoscopic retrograde cholangiopancreatography related perforations." HPB 17(0): 244.

23. Jin, Y.-J., et al. (2013). "Clinical course and proposed treatment strategy for ERCP-related duodenal perforation: a multicenter analysis." Endoscopy 45(10): 806-812.

24. Joshi, M. A., et al. (2017). "Treatment of duodenal peptic ulcer perforation by endoscopic clips: A novel approach." Journal of Digestive Endoscopy 8(01): 24-26.

25. Kaneko, T., et al. (1999). "Nonsurgical treatment of duodenal perforation by endoscopic repair using a clipping device." Gastrointestinal Endoscopy 50(3): 410-414.

26. Kessoku, T., et al. (2020). "Transformation of End-Stage Neuroendocrine Tumors With Uncontrollable Liver Metastasis Into a Novel or Additional Functional Phenotype." Frontiers in Oncology 10: 555963.

27. Khater, S., et al. (2017). "Over-the-scope clip (OTSC) reduces surgery rate in the management of iatrogenic gastrointestinal perforations." Endoscopy International Open 5(05): E389-E394.

28. Khokhar, O. S., et al. (2010). "Endoscopic management of iatrogenic duodenal perforation with linear-probe echoendoscope." Gastroenterology & hepatology 6(10): 657.

29. Kim, B. S., et al. (2011). "Management of endoscopic retrograde cholangiopancreatography-related perforations." Journal of the Korean Surgical Society 81(3): 195-204.

30. Kim, S. H., et al. (2019). "Endoscopic management of duodenal perforations caused by migrated biliary plastic stents." Endoscopy International Open 7(06): E792-E795.

31. Klein, A., et al. (2016). "Endoscopic mucosal resection of large and giant lateral spreading lesions of the duodenum: success, adverse events, and long-term outcomes." Gastrointestinal Endoscopy 84(4): 688-696.

32. Kocataş, A., et al. (2021). "Over-the-scope clip application for severe gastrointestinal bleeding, leak, or perforation: A single-center experience." Turkish Journal of Trauma and Emergency Surgery 27(1): 146.

33. Lee, T. H., et al. (2010). "Primary endoscopic approximation suture under cap-assisted endoscopy of an ERCP-induced duodenal perforation." World journal of gastroenterology: WJG 16(18): 2305.

34. Li, X.-Y., et al. (2020). "Application of endoscopic submucosal dissection in duodenal space-occupying lesions." World Journal of Clinical Cases 8(24): 6296.

35. Li, Y.-Y. Y., J.; Zhou, X.-J.; Chen, Y.-X.; Lv, N.-H.; Li, G.-H. (2019). "Management and outcomes of forty-five patients with ERCP-related perforations." Journal of Digestive Diseases 20(0): 90-91.

36. Li, Z., et al. (2021). "The value of endoscopic resection for non-ampullary duodenal lesions: A single-center experience." Saudi Journal of Gastroenterology: Official Journal of the Saudi Gastroenterology Association 27(5): 302.

37. Liu, Y., et al. (2016). "Endoscopic closure for EUS and ERCP related duodenal perforation by endoclips." Gastroenterology Research and Practice 2016.

38. Loske, G., et al. (2019). "Endoscopic negative pressure therapy (ENPT) for duodenal leakage–novel repair technique using open-pore film (OFD) and polyurethane-foam drainages (OPD)." Endoscopy International Open 7(11): E1424-E1431.

39. Mangiavillano, B., et al. (2016). "Over-the-scope clips in the treatment of gastrointestinal tract iatrogenic perforation: A multicenter retrospective study and a classification of gastrointestinal tract perforations." World Journal of Gastrointestinal Surgery 8(4): 315.

40. Miura, Y., et al. (2017). "Duodenal endoscopic submucosal dissection is feasible using the pocket-creation method." Endoscopy 49(01): 8-14.

41. Moyer, M. T., et al. (2019). "Transluminal washout and debridement of extraluminal contamination as an adjunct to endoscopic defect repair." VideoGIE 4(2): 91-94.

42. Nasa, M., et al. (2016). "Over-the-scope clip placement for closure of gastrointestinal fistula, postoperative leaks and refractory gastrointestinal bleed." Indian Journal of Gastroenterology 35: 361-365.

43. Nasa, M., et al. (2016). "Over-the-scope clip placement for closure of gastrointestinal fistula, postoperative leaks and refractory gastrointestinal bleed." Indian Journal of Gastroenterology 35: 361-365.

44. Nedoluzhko, I. Y., et al. (2019). "Endoscopic papillectomy for tumors of the major duodenal papilla." Annaly khirurgicheskoy gepatologii= Annals of HPB Surgery 24(1): 36-42.

45. Nishiyama, N., et al. (2013). "Efficacy and safety of over-the-scope clip: including complications after endoscopic submucosal dissection." World journal of gastroenterology: WJG 19(18): 2752.

46. Odemis, B., et al. (2016). "Can a fully covered self-expandable metallic stent be used temporarily for the management of duodenal retroperitoneal perforation during ERCP as a part of conservative therapy?" Surgical Laparoscopy, Endoscopy & Percutaneous Techniques 26(1): e9-e17.

47. Ojima, T., et al. (2018). "Laparoscopic and endoscopic cooperative surgery versus endoscopic submucosal dissection for the treatment of low-risk tumors of the duodenum." Journal of Gastrointestinal Surgery 22: 935-940.

48. Park, S. M., et al. (2015). "Feasibility of endoscopic resection for sessile nonampullary duodenal tumors: a multicenter retrospective study." Gastroenterology Research and Practice 2015.

49. Parlak, E., et al. (2013). "Endoscopic closure of ERCP-related duodenal perforations by using endoclips: a case series." Surgical Laparoscopy Endoscopy & Percutaneous Techniques 23(6): e225-e228.

50. Parlak, E., et al. (2020). "Fully covered self-expandable metal stens eliminate surgical repair requirement in both endoscopic sphincterotomy and precut sphincterotomy-related perforation (with video)." European Journal of Gastroenterology & Hepatology 32(5): 557-562.

51. Parodi, A., et al. (2010). "Endoscopic management of GI perforations with a new over-the-scope clip device (with videos)." Gastrointestinal Endoscopy 72(4): 881-886.

52. Raithel, M., et al. (2017). "Outcome, comorbidity, hospitalization and 30-day mortality after closure of acute perforations and postoperative anastomotic leaks by the over-the-scope clip (OTSC) in an unselected cohort of patients." Surgical Endoscopy 31: 2411-2425.

53. Santos, A. L., et al. (2020). "Endoscopic closure of lateral duodenal wall perforations caused by displacement of plastic biliary stents." Endoscopy 52(10): E357-E358.

54. Sbrozzi-Vanni, A., et al. (2020). "‘OVER THE SCOPE TECHNIQUE’(OTS) FOR PARTIALLY COVERED SELF-EXPANDABLE METAL STENT (PC-SEMS) PLACEMENT TO TREAT DUODENAL PERFORATION (DP) OCCURRED DURING EUS: CASE SERIES." Endoscopy 52(S 01): eP211.

55. Seo, J. Y., et al. (2014). "Usefulness and safety of endoscopic treatment for nonampullary duodenal adenoma and adenocarcinoma." Journal of Gastroenterology and Hepatology 29(9): 1692-1698.

56. Stassen, P. M., et al. (2021). "Prevalence of and risk factors for stent migration-induced duodenal perforation." Endoscopy International Open 9(03): E461-E469.

57. Takimoto, K., et al. (2022). "Efficacy of polyglycolic acid sheeting with fibrin glue for perforations related to gastrointestinal endoscopic procedures: a multicenter retrospective cohort study." Surgical Endoscopy 36(7): 5084-5093.

58. Tavusbay, C., et al. (2016). "Management of perforation after endoscopic retrograde cholangiopancreatography."

59. Thapa, N., et al. (2020). "Endoscopic or conservative management of iatrogenic duodenal perforations caused by long plastic biliary stent distal migration." ACG Case Reports Journal 7(7).

60. Theopistos, V., et al. (2018). "Non-operative management of type 2 ERCP-related retroperitoneal duodenal perforations: a 9-year experience from a single center." Gastroenterology research 11(3): 207.

61. Tringali, A., et al. (2018). "Temporary FC-SEMS for type II ERCP-related perforations: a case series from two referral centers and review of the literature." Scandinavian Journal of Gastroenterology 53(6): 760-767.

62. Valli, P. V., et al. (2017). "Nonampullary duodenal adenomas rarely recur after complete endoscopic resection: a Swiss experience including a literature review." Digestion 96(3): 149-157.

63. Voermans, R. P., et al. (2012). "Efficacy of endoscopic closure of acute perforations of the gastrointestinal tract." Clinical Gastroenterology and Hepatology 10(6): 603-608.

64. Wang, X., et al. (2020). "Duodenal perforations secondary to a migrated biliary plastic stent successfully treated by endoscope: case-report and review of the literature." BMC gastroenterology 20(1): 1-7.

65. Wang, Z.-Z., et al. (2021). "Effectiveness and safety of over-the-scope clip in closing perforations after duodenal surgery." World journal of gastroenterology 27(35): 5958.

66. Wedi, E., et al. (2016). "One hundred and one over-the-scope-clip applications for severe gastrointestinal bleeding, leaks and fistulas." World journal of gastroenterology 22(5): 1844.

67. Wichmann, D., et al. (2021). "Endoscopic negative pressure therapy as stand-alone treatment for perforated duodenal diverticulum: presentation of two cases." BMC gastroenterology 21: 1-6.

68. Yahagi, N., et al. (2018). "Outcomes of endoscopic resection for superficial duodenal epithelial neoplasia." Gastrointestinal Endoscopy 88(4): 676-682.

69. Zeng, C.-Y., et al. (2015). "Single-channel endoscopic closure of large endoscopy-related perforations." Endoscopy: 735-738.

70. Zhu, S., et al. (2019). "Purse-string sutures using novel endoloops and repositionable clips for the closure of large iatrogenic duodenal perforations with single-channel endoscope: a multicenter study." Surgical Endoscopy 33: 1319-1325.

71. Hoibian S, et al. (2021). “Endoscopic mucosal resection of sporadic duodenal nonampullary adenoma: outcomes of 130 patients with a long-term follow up in two tertiary French centers.” Ann Gastroenterol 34(2):169-176.
